# Myringoplasty in children: retrospective analysis of 35 cases

**DOI:** 10.1016/S1808-8694(15)30654-6

**Published:** 2015-10-19

**Authors:** Nemer Al-Khtoum, Mohammad Ali Hiari

**Affiliations:** 1 Otorhinolaryngologist (senior Otorhinolaryngologist at king Hussein Medical Center, Royal Medical Services (Amman-Jordan)); 2 Otorhinolaryngologist ((senior Otorhinolaryngologist at king Hussein Medical Center, Royal Medical Services (Amman-Jordan)) Department of Otolaryngology, King Hussein Medical Center (Amman- Jordan)

**Keywords:** hearing, myringoplasty, tympanic membrane perforation

## Abstract

**Aims:** to carry out a retrospective analysis of myringoplasty results in children in our institute.

**Materials and methods:**

Thirty five children, 9 to 14 years old, who underwent myringoplasty in our hospital between April 2002 and May 2004, formed the study group. Data regarding successful perforation closure, factors influencing success rates and hearing improvement were recorded.

**Results:**

Closure of perforation was successful in 30 (85.7%) of the 35 patients. Graft take failure occurred in 5 patients. Audiological improvement was seen in 27 (77%) patients, out of which 23 cases had 10-15 db and 4 cases had 15-20 db air-bone gap. Hearing was found to be worse postoperatively in 3 patients, while no change was noted in the remaining 5 patients. There was no case of profound hearing loss.

**Conclusion:**

Myringoplasty is a beneficial procedure in the pediatric population in the hands of a skilled and experienced surgeons. If performed properly, it has a good chance of restoring a child's hearing. However, a large study with a long follow up is warranted in order to come to a definitive conclusion.

## INTRODUCTION

Otologic surgery in children is regarded by many as being less successful than in adult patients. The higher incidence of otitis media in the pediatric population is often implicated as the reason for poorer results. This leads to disparate opinions concerning the appropriate indications for tympanoplasty in children. Most would agree that the ear with cholesteatoma or some other middle ear tumor warrants surgery. The chronically draining ear that is resistant to medical therapy also requires surgery. However, the management of patients with persistent perforation of the tympanic membrane (TM), with or without intermittent otorrhea, incites considerable controversy. Some advocate early surgery to correct anatomic defects and improve hearing. Others maintain elective surgery should be deferred until the peak incidence of acute otitis media has passed.

Pediatric myringoplasties were performed as early as 1962 in the United States^1^ and in the early 1970s in the United Kingdom.^2^ Since then, several studies on pediatric myringoplasty published in English have reported success rates between 56% and 94%.^3^ Criteria of success and study design were not similar in these studies, making it difficult to compare results and draw impartial conclusions. Nevertheless, a meta-analysis concluded that there was no difference associated with age in the success rate of myringoplasty.^3^

The aim of present study was to carry out a retrospective analysis of the results of myringoplasty in children in our institute.

## MATERIAL AND METHODS

After approval from institutional Ethical Committee of Royal Medical Services, Amman- Jordan (approval protocol number: RMS/07/032/07).

Thirty five children, 9 to 14 years old, who underwent myringoplasty in the department of Otolaryngology at King Hussein Medical Center in Jordan between April 2002 and May 2004, formed the study group. The mean age at the time of operation was 12 years.

All the children had central perforations that had remained dry for a minimum period of 10 weeks with a good cochlear reserve as assessed by preoperative pure tone audiometry.

Patients who underwent any type of mastoidectomy on the same operative date or in the same ear at a previous date, who had any type of previous or concurrent ossicular chain reconstruction, or who had no follow-up data available were excluded from the study.

Data regarding successful closure of perforation, factors influencing success rate and hearing improvement were recorded.

Successful closure of perforation was defined as an intact eardrum at 1 year postoperatively. Success in terms of hearing was defined as an improvement of 10 dB or greater in 2 consecutive frequencies compared with the preoperative air conduction thresholds.^4^ Preoperative and postoperative thresholds were measured at 500, 1000, 2000 and 4000 Hz.

Surgery was performed by a senior surgeon. An endaural approach was used in all cases. The temporalis fascia graft was harvested and positioned medial to the drum remnant using the underlay technique.

Hospital stay is of one day, using oral analgesic and IV antibiotics, keeping oral medication for one week after hospital discharge.

## RESULTS

We have noticed that success rate was slightly better in 12-14 years age group as compared to age group 9-11 years.

Closure of perforation was successful in 30 (85.7%) of the 35 patients. Failure of the graft occurred in 5 patients.

Audiological improvement was seen in 27 (77%) patients out of whom 23 cases had 10-15 db air-bone gap (mean preoperative and postoperative air-bone gap27.4 ± 7.6 vs 11.4 ± 6.1 dB respectively).

Four cases had 15-20 db air-bone gap (mean preoperative and postoperative air-bone gap 30.4 ± 4.6 vs 17.4 ± 5.1 dB respectively).

Hearing was found to be worse postoperatively in 3 patients (mean preoperative and postoperative air-bone gap 26.7 ± 5.8 vs 34.6 ± 7.2 dB respectively), while no change was noted in the remaining 5 patients. There was no case of profound hearing loss.

Other factors that contributed in the outcome of myringoplasty were duration of ear discharge, period of inactivity, size of perforation, status of contralateral ear and condition of middle ear mucosa (Table I). We noticed a higher success rate when there is short duration of ear discharge, longer period of dry ear and the small size of perforation.

**Table 1:** Factors influencing success rate of Myringoplasty.

Contributing factors	No. of Cases	Success Rate (%)	p value
**Age**			
12-14 years	20	87,4	>0,05
9 −11 years	15	83,9	
**Duration of ear discharge**			
1-2 years	17	90,2	>0,05
3-5 years	11	84,8	
6-9 years	7	81,9	
**Duration of dry ear**			
>15 weeks	15	93,3	<0,05
13-15 weeks	12	83,9	
10-12 weeks	8	79,7	
**Size of perforation**			
Small central	23	94,7	<0,01
Subtotal	12	66,9	
**Status of Contralateral ear**			
Normal	25	87,6	>0,05
Perforated	10	83,7	

Out of 5 cases that had failed, 3 cases were found to have edematous or inflamed middle ear mucosa at the time of surgery.

Also, out of these five cases that had failed, four cases had had complete graft rejection and 2 out of it showed deterioration of the hearing level while the remaining 2 showed no change in the hearing level. One case was left with a small residual perforation with no change in hearing level.

## DISCUSSION

Perforation of the tympanic membrane in children can cause significant disability. Myringoplasty is a simple and effective procedure that results in the successful closure of the perforation in most cases. However, there seems to be no consensus among otologists regarding the benefits of myringoplasty in children.^5^, ^6^, ^7^ The rationale for operating early in children is 3-fold^6^: (1) to prevent the possibility of chronic ear disease and its related complications; (2) to improve hearing without the need for a hearing aid and thus optimize one of the main conditions for speech and language development; and (3) to help the child enjoy water activities. On the other hand, persistent eustachian tube (ET) dysfunction, recurrent upper respiratory tract infections, technical difficulty, and reperforation are the predominant arguments put forward for delaying the procedure until a certain age,^7^ which can vary from 10 to 14 years. It has also been argued that a perforation in the eardrum is an equivalent to a ventilation tube.^5^

Timing of repair in the pediatric population is very controversial. Glasscock^9^ gave young age as a relative contraindication to tympanoplasty because children under three or four are prone to upper respiratory infections and otitis media. Koch et al^5^ reported an 81% success rate for children age 8 and older, but only a 30% success rate in younger patients. They concluded that tympanoplasty before age 8 results in a high rate of failure because of poor Eustachian tube function and frequent URIs. Smyth^10^ agreed, noting that patients less than 10 years old had a higher failure rate for myringoplasty than older children. This was independent of secretion type, perforation site, and graft material.

Yet, others such as Lau and Tos^11^ found no significant difference in outcome between the 2 to 7 age group and those children ages 8 to 14. They suggested that early operation may prevent progression of ossicular chain resorption. Ophir et al^12^ reported a 79% overall success rate, and their success in younger children (5-8) was comparable to the rate for older children. They concluded that myringoplasty had a good chance of success at any age. Kessler et al^6^ reviewed the results of 209 myringoplasties and concluded that even in young patients (2-6 years) myringoplasty has a high success rate (75-94%), and that age alone could not be considered a contraindication to surgery.

The outcome of myringoplasty depends on the criteria for selection and the length of follow-up. If closure of perforation alone is taken as a measure of success, the rate is reported to be between 75% and 92%.12 This compares favorably with the results reported for the adult population.^13^, ^14^ However, success rate can be as low as 45% if factors such as occurrence of OME, reinsertion of ventilation tubes, and atelectasis are considered measures of failure.^5^, ^12^ Although enjoyment of water activities and absence of otorrhea are well-recognized benefits that improve the quality of life of children after myringoplasty, there is as yet no scale to measure these benefits. It is therefore crucial to define the criteria of success in pediatric myringoplasty, preferably internationally, to enable us to compare the results in a more meaningful way.

The present study was conducted on patients aged 9-14 years and the result of successful graft uptake was 85.7% which is comparable to the results of various authors.^4^, ^5^, ^15^, ^16^

The reason for variation in results of these authors could be attributed to the wide range of age that differs in various studies, because of technique used, varying length of follow up and experience of surgeons.

The post operative air-bone gap was less than 20 db in our study in 77% of cases comparable to other authors. ^16^,^17^ while no change in hearing was noted in 5 patients.

We were unable to verify the cause of deterioration of the hearing level that was noted in 3 patients.

## CONCLUSION

Myringoplasty is a beneficial procedure in the pediatric population in the hands of a skilled and experienced surgeon. If performed properly, stands a good chance of restoration of hearing in children. However, a large study with a long follow up is warranted to come to a definite conclusion.
